# Systematic Review and Meta‐Analysis of Resection Sequence in Synchronous Colorectal Liver Metastasis: Primary vs. Liver First

**DOI:** 10.1002/jso.70309

**Published:** 2026-06-15

**Authors:** Sofoklis Mavrantonis, Omar Abou El‐Wafa, Sascha Vaghiri, Dimitrios Prassas

**Affiliations:** ^1^ General Surgery Service Gloucestershire Hospitals NHS Foundation Trust Gloucester UK; ^2^ Department of Surgery (A) Heinrich‐Heine‐University, Medical Faculty and University Hospital Duesseldorf Duesseldorf Germany; ^3^ Department of Surgery Katholisches Klinikum Essen, Philippusstift, Teaching Hospital of Duisburg‐Essen University Essen Germany

## Abstract

**Background:**

This present systematic review and meta‐analysis aims to compare overall survival (OS), disease‐free survival (DFS), morbidity and mortality outcomes in patients undergoing colorectal first (CRf) versus liver first (Lf) resections with synchronous colorectal liver metastases.

**Methodology:**

A literature search was performed in PubMed, Cochrane Central trials register, and Google Scholar databases. This was conducted in line with current PRISMA guidelines. 95% confidence intervals were calculated for the primary endpoints and odds ratios for secondary endpoints. The Cochrane *Q* test and the measurement of inconsistency test were used to assess the degree of heterogeneity among the included studies.

**Results:**

Fifteen studies were included with a total of 8611 patients from 2010 to 2025. A statistically significant survival benefit was seen in the overall 1‐year survival of the CRf group compared to the Lf group, with no significant difference in 3‐ and 5‐year follow‐up. In addition, a statistically significant benefit in 1‐, 3‐, and 5‐ year DFS rates was seen in the CRf group when compared to the Lf group. No significant difference was seen in major morbidity and 90‐day mortality, and failure to proceed to the second resection stage.

**Conclusion:**

DFS was significantly increased in the CRf group compared to the Lf group, with no difference in secondary outcomes. One‐year OS was also found to be higher in the CRf group; however, this finding likely reflects the heterogeneity of the included studies and perioperative course after hepatic resections. To our knowledge, this is the first meta‐analysis to demonstrate such a significant difference of one strategy over the other. However, caution should be used in the interpretation of these results due to the lack of available randomized control trials.

## 1 Introduction

Colorectal cancer (CRC) remains the second most common cancer in Europe and accounts for 12.7% of all new cancer diagnoses and 12.4% of all malignancy‐related deaths [1, 2]. Due to anatomical factors of the colonic and liver circulation, there is a high incidence of colorectal tumors that metastasize in the liver [3], with approximately 20% of CRC patients presenting with simultaneous colorectal liver metastases (CRLM) [4]. It has been well described that synchronous disease carries a significantly worse prognosis than metachronous disease, and resection of both tumor sites is mandatory for curative treatment [5, 6]. There are three main operative strategies: (1) Primary first, where the colorectal tumor is resected followed by liver resection, (2) Liver first, where the colorectal resection is done second, and (3) Simultaneous resection. Further treatment adjuncts, such as perioperative chemotherapy and radiotherapy, are guided through a multi‐disciplinary team (MDT) approach and should be tailored to individual patients.

It is important to note that no strategy so far has shown a significant difference in outcomes between the three strategies, with MDT outcomes, institutional policy, and subspecialty availability guiding resection strategy [7]. In addition, patient selection in studies evaluating the above strategies is key, with patients undergoing simultaneous resection requiring a higher pre‐operative physiological reserve than their staged counterparts. Another challenge posed in studies evaluating staged resections is the proportion of patients who failed to proceed to the second stage, with per‐protocol analyses carried out, making comparisons more prone to errors.

There are several studies evaluating simultaneous versus sequential approaches; however, evidence regarding liver‐first approaches versus primary first approaches is sparser [8, 9, 10, 11]. Despite this, clear evidence supporting the selection of the simultaneous strategy is lacking, with similar overall survival rates to the staged groups, as most of it derives from cohort studies. The recent METASYNC randomized controlled trial (RCT) was the first to demonstrate a trend toward improved overall survival (OS) and disease‐free survival (DFS), providing support for a simultaneous resection strategy. Notably, the staged resection arm of this trial explicitly included patients managed with a colorectal‐first (CRf) approach [12].

In terms of evidence comparing liver‐first versus primary‐first approaches, challenges are posed by the lack of RCTs, with most studies being mostly retrospective observational studies along with a lack of meta‐analyses. One meta‐analysis published in 2020 by Magouliotis et al., comparing liver versus primary first approach, did not show any clear superiority of one strategy over the other; however, the above challenges of available evidence remain [13].

In this study, we aim to conduct a systematic review and meta‐analysis of primary literature comparing outcomes between primary versus liver‐first resection strategies for CRLM. We will specifically focus on pooled analysis of morbidity, mortality, and overall (OS) and disease‐free survival (DFS) rates in these cohorts.

## 2 Materials and Methods

The present work was conducted in accordance with the current PRISMA (Preferred Reporting Items for Systematic Reviews and Meta‐Analyses) statement and the latest version of the Cochrane Handbook for Systematic Reviews of Interventions, to ensure transparency and adequate study evidence [14, 15]. This meta‐analysis was registered with PROSPERO (ID: CRD420261278670).

### 2.1 Literature Search

Two authors (S.V. and O.A.) independently performed a comprehensive literature review in the PubMed (Medline), the Cochrane Central trials register, and Google Scholar databases until May 1st, 2025, through screening of article titles and abstracts. No language restriction was applied. The following medical subject headings were combined with the Boolean operators AND or OR: [(colorectal liver metastases) or (colorectal hepatic metastases) or (synchronous colorectal liver metastases) or (liver resection) or (liver‐first) or (staged surgery) or (reverse strategy) or (primary tumor) or (classical strategy) or (bowel‐first)] and [(colorectal cancer)]. Disagreements were resolved by discussion and consensus or consultation of a third author (D.P.) if necessary.

### 2.2 Inclusion and Exclusion Criteria

All comparative studies reporting clinical outcomes of liver‐first versus colorectal‐first approach for synchronous hepatic metastatic disease were included. Two‐armed studies including simultaneous resections were not included. The exclusion criteria were as follows: case/technical reports, editorials or narrative reviews, non‐peer‐reviewed articles, studies failing to demonstrate the number of cases lost‐to follow up, studies from which the censored cases at follow‐up could not be extracted, and studies that did not report specific outcomes of interest. For data integrity, the reported outcomes of interest in each included study should enable the calculation of odds ratios (ORs) with 95% confidence intervals (CIs). Only studies with adult participants were considered. Discrepancies in study selection were resolved either by consensus or by consultation with an independent third author (D.P.).

### 2.3 Data Extraction and Outcomes of Interest

Two authors (S.M. and O.A.) independently collected all available and relevant data from studies meeting the inclusion criteria. The extracted data contain the following information: (a) study characteristics (authors, year and country of publication, enrollment period, study design and protocol, number of included patients), (b) patients' baseline demographics. comorbidities, disease characteristics, and data on adjuvant therapy, (c) surgical technique and intraoperative details, and (d) postoperative short‐ and long‐term outcomes. If the authors were unable to achieve consistent results during data extraction, an independent third reviewer (D.P.) was consulted for advice. The primary endpoint was 1‐, 3‐, and 5‐year overall survival (OS) as well as disease‐free survival (DFS). The secondary outcomes included major complications after liver metastasis and primary first resection (according to Clavien‐Dindo and ACS‐NSQIP, respectively [16, 17], 90‐day mortality, and failure to proceed to the second stage.

### 2.4 Quality and Certainty Assessment

The ROBINS‐I tool was used to assess the risk of bias of the included non‐randomized trials, as recommended in the Cochrane Handbook for Systematic Reviews of Interventions [18]. This tool categorizes the risk of bias in non‐randomized studies from low to critical based on seven potential bias domains. The revised AMSTAR 2 instrument was used to critically appraise this meta‐analysis [18]. The level of evidence for the significant outcomes was categorized into four classes according to GRADE (The Grading of Recommendations, Assessment, Development, and Evaluation) [19]. Study quality and certainty judgment was performed independently by two authors (O.A. and S.M.). Disagreements during this step were discussed and resolved by consensus or reassessment by a third author (D.P.).

### 2.5 Statistical Analyses

Data of interest were analyzed with pairwise meta‐analyses. For each primary and secondary outcome, summary treatment effect estimates with 95% CIs were calculated. For dichotomous endpoints, the odds ratio was chosen as the effect measure. Using the Cochrane *Q* test (*χ* ^2^ test) and the measurement of inconsistency (*I* ^2^), the degree of heterogeneity among the included studies was interpreted as follows: 0%–40% low heterogeneity and may not be important, 30%–60% moderate heterogeneity, 50%–90% substantial heterogeneity, ≥ 75% considerable heterogeneity [14]. When heterogeneity was low

or moderate (*I* ^2^ ≤ 50%), summary estimates were calculated using a fixed‐effects method. Where appropriate, subgroup analysis was conducted to ensure the robustness of the overall heterogeneity in the results. Leave‐one‐out analysis investigated the effect of each study on the pooled outcomes. Statistical significance was defined as *p* values ≤ 0.05 of pooled data. Statistical analysis was performed using RevMan software (version 5.3; Copenhagen: The Nordic Cochrane Centre, The Cochrane Collaboration, 2014).

## 3 Results

### 3.1 Study and Patient Characteristics

**Figure 1:**
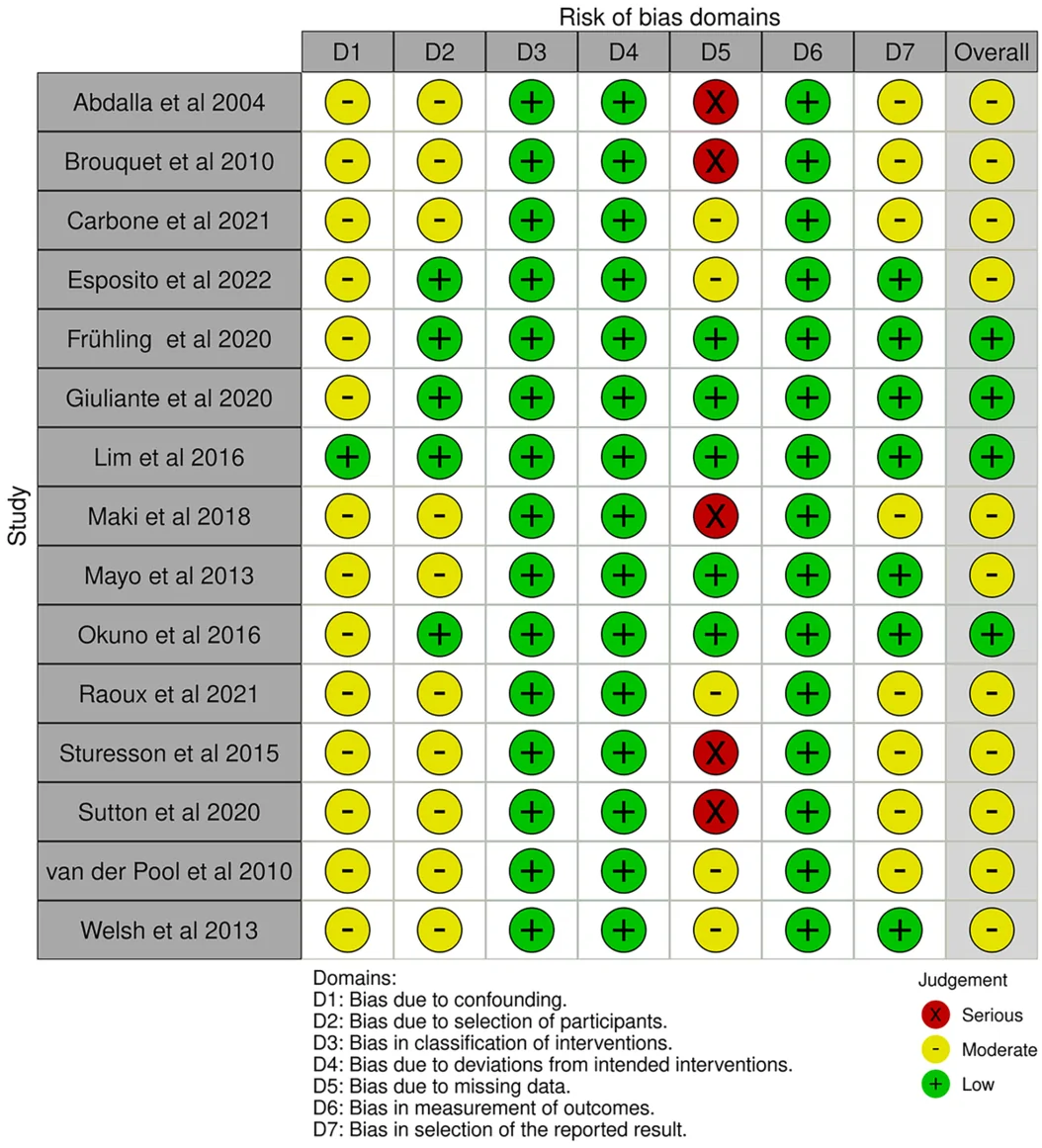
Robins plots indicating risk of bias in included studies.

Our initial systematic database search identified 27 845 records. After removing duplicates and irrelevant articles, 29 full‐text articles were assessed for eligibility. Based on the predefined inclusion criteria, 15 studies [20, 21, 22, 23, 24, 25, 26, 27, 28, 29, 30, 31, 32, 33, 34] were eligible for our final meta‐analysis (Figure 1).

All of the included studies were retrospective. A total of 8611 patients were enrolled from 2010 to 2025 (CRf: *n* = 7296, Lf: *n* = 1315). Included studies encompassed primary tumor sites of colon and rectum. Three studies analyzed exclusively cases with rectal cancer (Maki, Stureson, van der Pool). A summary of the study, patient, and surgical characteristics is presented in Table 1.

**Table 1:** Study characteristics, indicating numbers of patients in the classical (C) and reverse (R) groups, along with proportions of rectal and colonic primaries. Patients in simultaneous resection groups were not included in the analysis.

Author (et al.)	Year	Country	Design	Period	N per group	Primary site	Total N	Comparison	Citation
Abdalla et al.	2025	France	Retrospective multicentric cohort	2011–2020	C: 40, R: 31, S:12	Rectum 100%	71	Rectum‐first vs. Liver‐first vs. Simultaneous	*Surgery* 2025; 181:109291
Carbone et al.	2022	UK	Retrospective single‐center cohort	2009–2019	C: 129, R: 26, S: 49	Rectum**:** R: 13 (50%). C: 21 (16.3%) Left colon: R: 5 (19.2%). C: 10 (7.8%) Ascending colon: R: 2 (7.7%), C: 41 (31.8%) Sigmoid: R: 6 (23.1). C: 57 (44.1%)	155	Primary‐first vs. Liver‐first vs. Simultaneous	*Updates Surg* 2022; 74:451–465
Sutton et al.	2025	USA	Retrospective propensity‐matched cohort	2003–2019	C: 93, R: 35, S: 23	Rectal/Rectosigmoid: C + S: 24 (20.7%). R: 31 (88.6%)Sigmoid/Descending: C + S: 47 (40.5%). R: 4 (11.4%) Ascending/Cecum: C + S: 36 (31%). R: 0 (0%)	128	Liver‐first vs. Primary‐first/Simultaneous	*Dis Colon Rectum* 2025; 68:32–40
van der Pool et al.	2010	Netherlands	Retrospective single‐center cohort	2000–2007	C: 29, R: 20, S:8	Rectum 100%	49	Primary‐first vs. Simultaneous vs. Liver‐first	*Br J Surg* 2010; 97:383–390
Welsh et al.	2016	UK	Retrospective propensity‐matched cohort	2004–2014	C: 467, R: 98	Rectum: C: 130 (27.8%). R: 44 (45%) Colon: C: 337 (72.2%). R 54 (55%).	565	Liver‐first vs. Classical	*Br J Surg* 2016; 103:600–606
Brouquet et al.	2010	USA	Retrospective cohort	1992–2009	C: 72, R: 27, S: 43	Rectum: C:35 (48.6%) R: 19 (70.4%) Colon: C:37 (51.4%). R: 8 (29.6%)	99	Classic vs. Combined vs. Reverse	*J Am Coll Surg* 2010; 210:934–941
Lim et al.	2016	France	Retrospective single‐center PSM cohort	1989–2013	C: 145, R: 16	Rectum: C: 29 (20%), R: 8 (50%) Colon: C: 116 (80%), R: 8 (50%)	161	Classical vs. Reverse	*Ann Surg Oncol* 2016; 23:3024–3032
Mayo et al.	2013	USA/Europe	Retrospective multicentric	1982–2011	C: 647, R: 27, S: 329	Rectum: C: 170 (26.4%). R: 15 (53.6%) Colon: C: 475 (73.6%). R 13 (46.4%)	674	Simultaneous vs. Classic vs. Liver‐first	*J Am Coll Surg* 2013; 216:707–718
Okuno et al.	2016	Japan	Retrospective single‐center	2006–2013	C: 13, R: 12	Rectum: C: 8 (61.6%). R: 7 (58.3%) Colon: C: 5 (38.4). R 5 (41.7%)	25	Liver‐first vs. Primary‐first	*Surg Today* 2016; 46:721–728
Esposito et al.	2023	UK	Retrospective single‐center	2009–2019	C: 587, R: 66	Rectum: C: 28 (44.4%) R: 27 (42.9%)Colon: C: 35 (55.6%). R: 36 (57.1%)	653	Primary‐first vs. Liver‐first vs. Simultaneous	*HPB* 2023; 75:451–465
Frühling et al.	2023	Sweden	Retrospective bi‐institutional cohort	2009–2020	CL 403, R: 163, S: 92	Rectum: C: 107 (27.6%). 108 (66.3%)Left colon: C: 182 (45.2%). R: 44 (27.0%)Transverse colon: C: 13 (3.2%) R: 2 (1.2%) Ascending colon: C: 94 (23.3%) R: 9 (5.5%)	566	Primary‐first vs. Liver‐first vs. Simultaneous	*HPB* 2023; 25:26–36
Giuliante et al.	2021	France	Registry‐based multicenter study	2000–2017	C: 4415, R: 552, S: 2393	Rectum: C: 1331 (34.7%). R: 317 (57.9%) Left Colon: C: 1994 (46.2%) R: 162 (26.6%) Right & Transverse Colon: C: 993 (22.9%) R: 68 (12.4%)	4967	Liver‐first vs. Primary‐first vs. Simultaneous	*Ann Surg* 2021; 273:1089–1099
Mäki et al.	2025	USA	Retrospective single‐center	2004–2021	C: 73, R: 141, S: 60	Rectum 100%	214	Reverse vs. Classical	*Ann Surg Oncol* 2025; in press
Raoux et al.	2025	France	Retrospective multicentric	2008–2022	C: 149, R: 26, S: 34	Rectum: C: 32 (21.5%). R:5 (19.2%)Colon: C: 117 (78.5%). R: 21 (80.8%)	175	Rectum‐first vs. Liver‐first vs. Simultaneous	*Surgery* 2025; 181:109291
Sturesson et al.	2017	Sweden	Nationwide retrospective cohort	2008–2015	C: 34, R: 75	Rectum: C: 15 (44.1%). R: 47 (62.6%) Colon: C: 19 (55.9%). R: 28 (37.3%)	109	Liver‐first vs. Classical	*HPB* 2017; 19:1–7
					Total C: 7296 Total R: 1315		8611		

### 3.2 Study Quality and Risk of Bias

**Figure 2:**
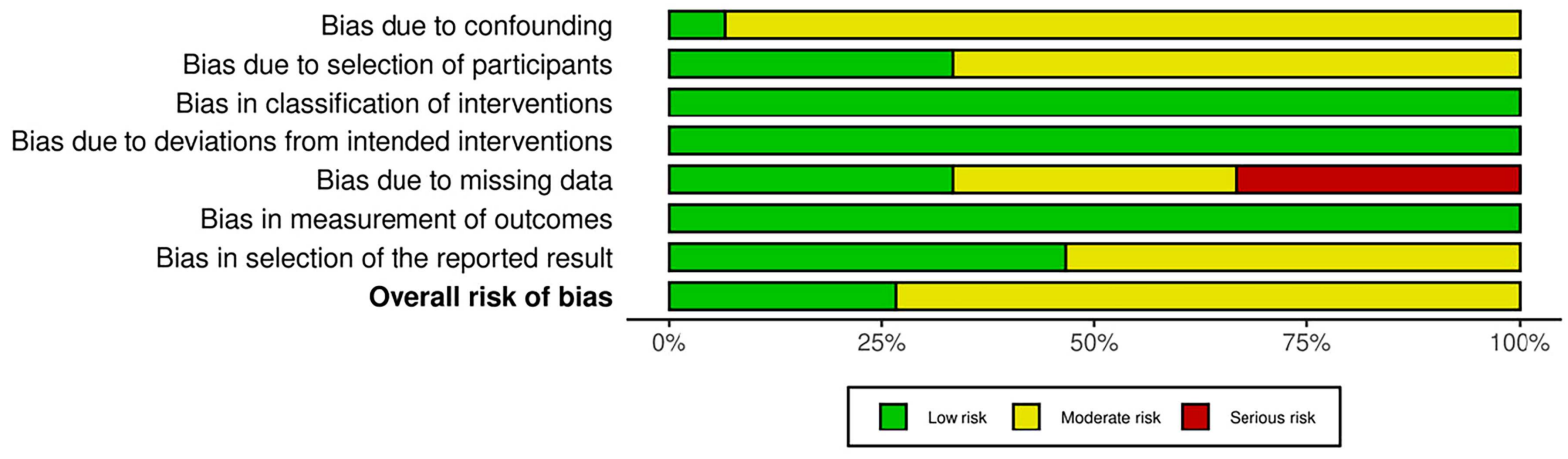
Proportion of degree of bias as related to identified causes.

The overall risk of bias in the 15 included studies was low to moderate (Figures 1 and 2). The methodological quality of the present meta‐analysis was determined as “high” using the AMSTAR 2 quality assessment tool. Of note, not all outcomes of interest were available in each study.

## 4 Primary Endpoints

### 4.1 OS

**Figure 3:**
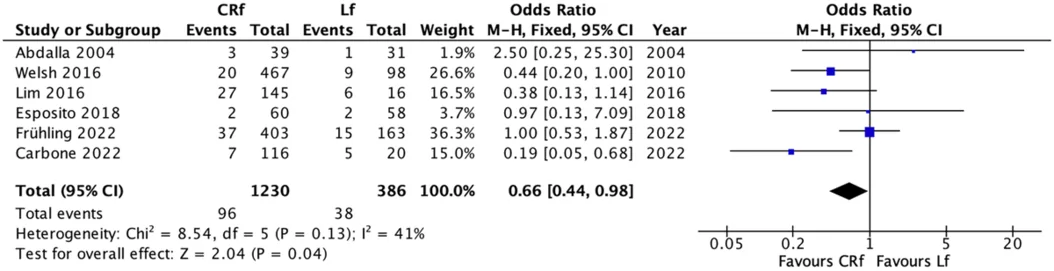
Forest plot describing the difference in OS between CRf and Lf at 1 year of follow‐up. Statistically significant benefit in the CRf strategy seen.

**Figure 4:**
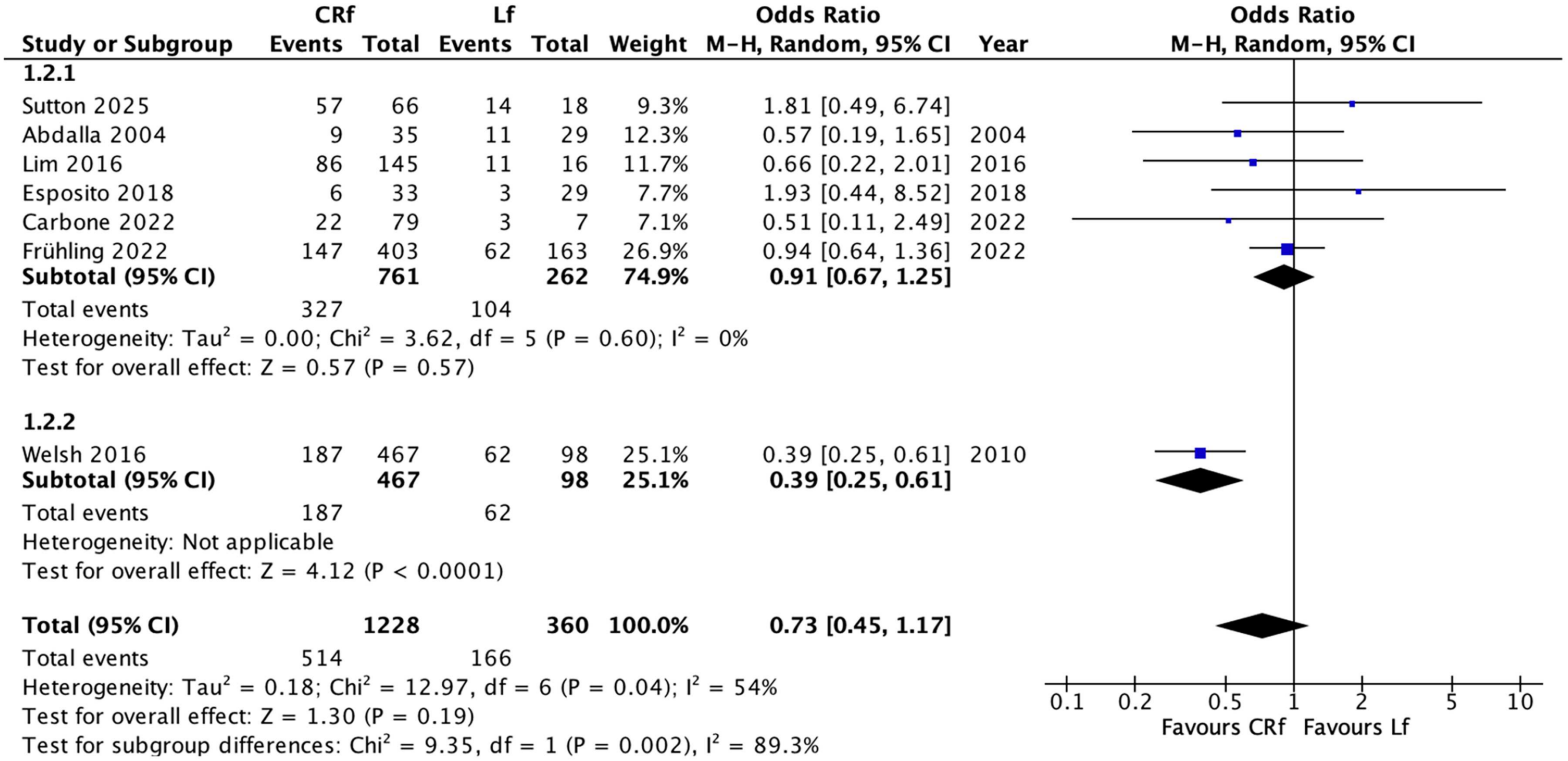
Forest plot describing the difference in OS at 3‐year follow‐up. The previously seen benefit in OS with the CRf strategy is no longer observed with the exclusion of Welsh et al due to higher heterogeneity.

**Figure 5:**
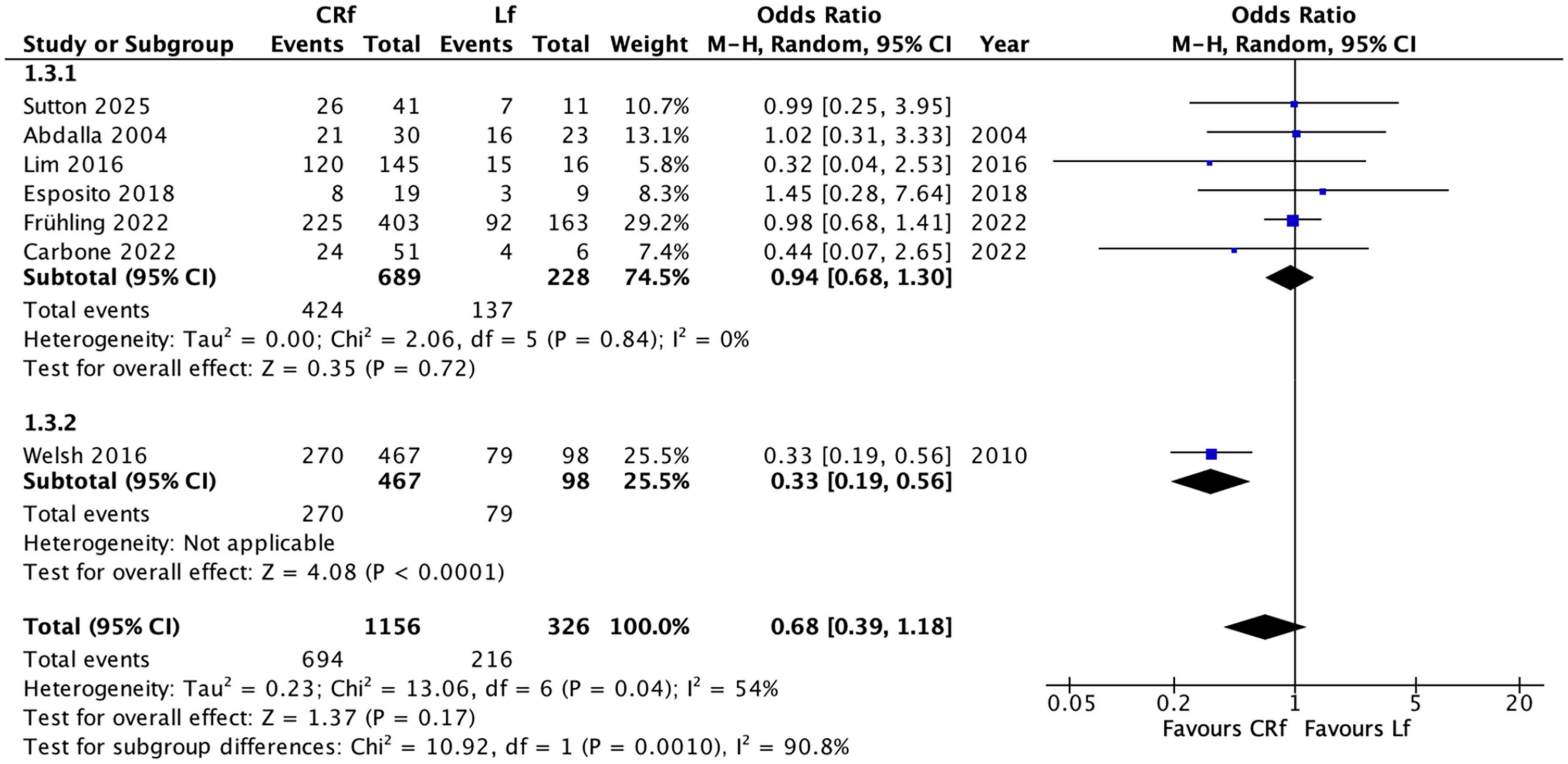
Forest plot describing the difference in OS at 5‐year follow‐up, with no statistically significant differences between the two groups.

Meta‐analysis demonstrated a statistically significant difference between the two groups regarding OS at 1 year in favor of the CRf subgroup (OR = 0.66; 95% CI 0.44–0.98; *p* = 0.04; *I* ^2^ = 41%) Figure 3. There were 1230 patients in the CRf group and 386 in the Lf group. The certainty of evidence was judged as high based on GRADE criteria (Table S1). This effect was eliminated at 3‐ and 5‐year follow up after excluding the study of Welsh et al. [24] as a recognized source of heterogeneity (OR = 0.91; 95% CI 0.67–1.25; *p* = 0.57; *I* ^2^ = 0) Figure 4 and (OR = 0.94; 95% CI 0.68‐–1.30; *p* = 0.72; *I* ^2^ = 0) Figure 5, respectively.

### 4.2 DFS

**Figure 6:**
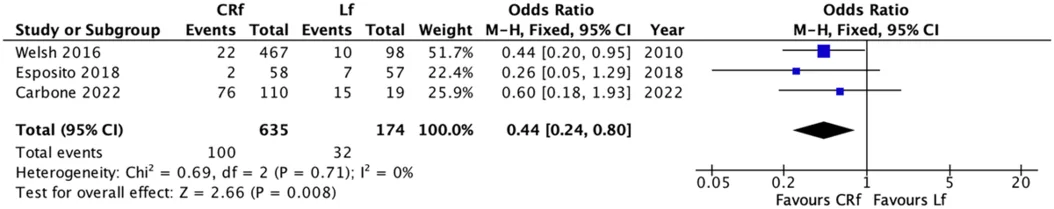
Forest plot demonstrating a statistically significant benefit in DFS in CRf compared to Lf strategy at 1‐year follow‐up.

**Figure 7:**
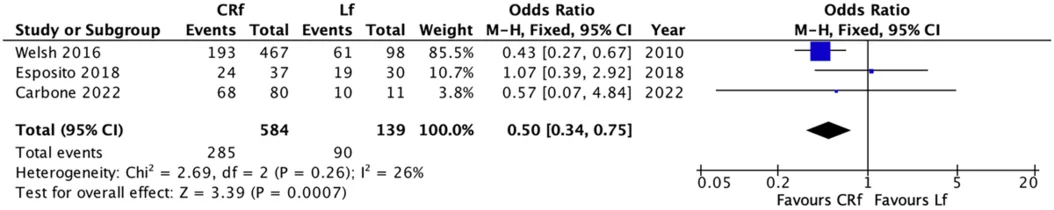
Forest plot demonstrating the sustained benefit seen above in DFS in the 3‐year follow‐up period.

**Figure 8:**
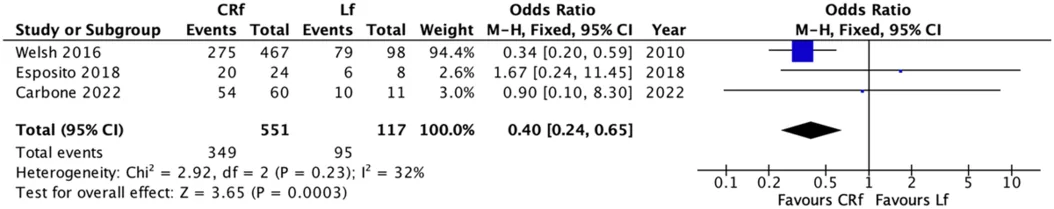
Forest plot demonstrating the sustained benefit seen above in DFS in the 5‐year follow‐up period.

Disease‐free survival was analyzed in three studies [21, 24, 29]. There were 635 patients in the CRf group, and 174 patients in the Lf group at 1‐year follow up, 584 and 139 at 3‐year follow‐up, and 551 and 117 patients at 5‐year follow up, respectively. The positive effect of the primary first approach was more evident in DFS. One‐, 3‐, and 5‐year DFS were significantly longer for the CRf subgroup with OR = 0.44; 95% CI 0.24–0.80; *p* = 0.008; *I* ^2^ = 0%, OR = 0.50; 95% CI 0.37–0.75; *p* < 0.001; *I* ^2^ = 26% and OR = 0.40; 95% CI 0.24–0.65; *p* < 0.001; *I* ^2^ = 32% respectively. The certainty of evidence was judged as moderate for each yearly interval, based on GRADE criteria (Table S1). Of note, only three studies reported on this primary endpoint (Figures 6, 7, 8).

## 5 Secondary Endpoints

### 5.1 90‐Day Mortality

**Figure 9:**
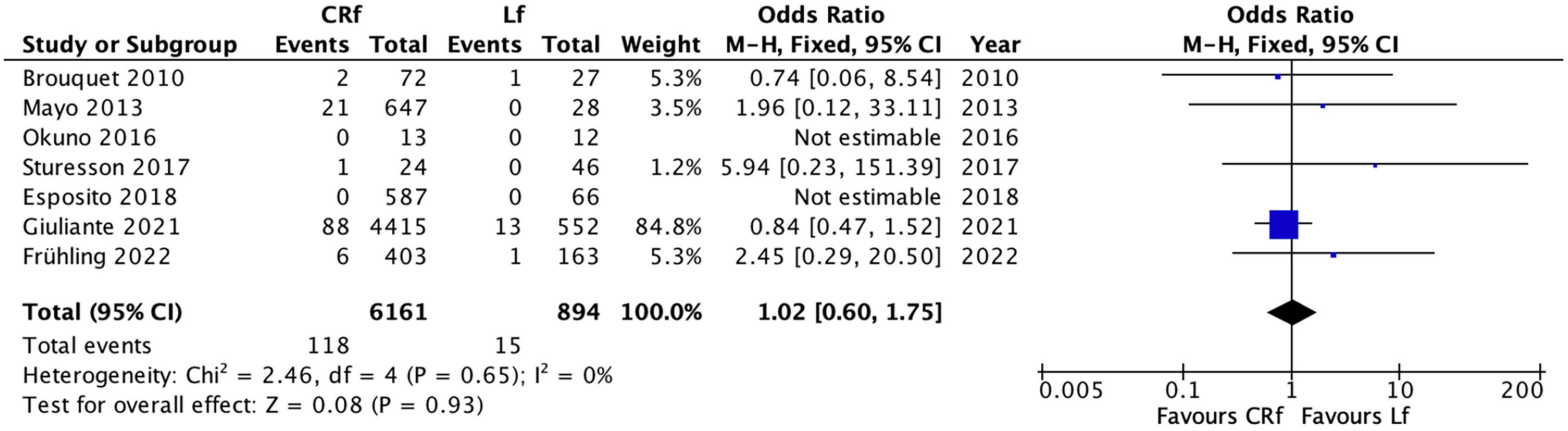
Forest plot demonstrating no significant difference in 90‐day post‐operatively mortality in the five studies that reported on this endpoint.

Seven studies reported on 90‐day Mortality [21, 27, 28, 29, 30, 31, 34], with two being non‐estimable. Upon meta‐analysis no significant difference was noted between the two study groups. (OR = 1.02; 95% CI 0.41–1.33; *p* = 0.93; *I* ^2^ = 0%) (Figure 9).

### 5.2 Failure to Proceed to the Next Stage

**Figure 10:**
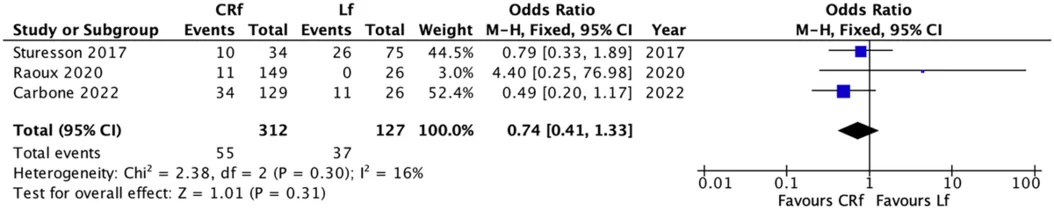
Forest plot demonstrating no significant difference in failure to proceed to the second resection.

Three studies reported on this outcome [21, 33, 34]. There was a combined total of 55 patients in the CRf that failed to proceed to the next resection stage, and 71 in the Lf group in these studies. No approach was found to significantly differ from the others (OR = 0.74; 95% CI 0.41–1.33; *p* = 0.31; *I* ^2^ = 16%) (Figure 10).

### 5.3 Major Morbidity

**Figure 11:**
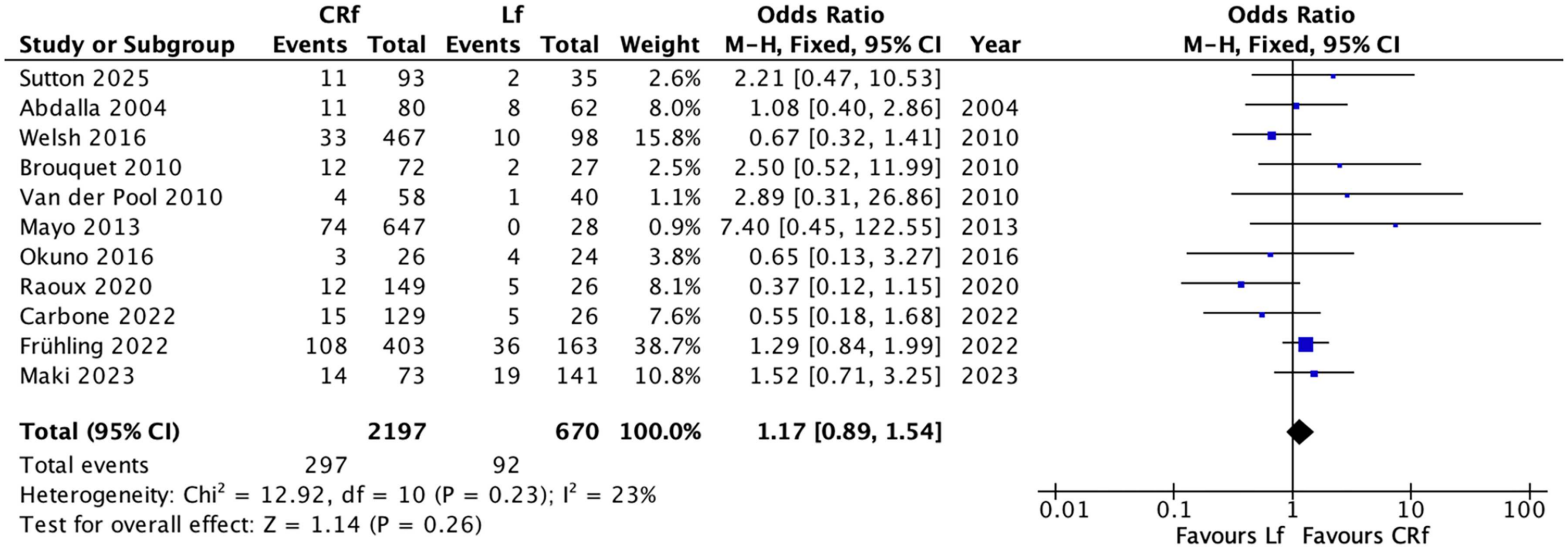
Forest plot comparing major morbidity as measured using the Clavien‐Dindo scale. No difference was demonstrated.

Eleven studies reported on major morbidity (Clavien‐Dindo ≥ IIIa) [20, 21, 22, 23, 24, 25, 27, 28, 30, 32, 33]. The rate of major postoperative morbidity was not statistically different between both subgroups. (OR = 1.17; 95% CI 0.89–1.54; *p* = 0.26; *I* ^2^ = 23%) (Figure 11).

## 6 Discussion

To our knowledge, this is the most recent systematic review and meta‐analysis comparing the primary first versus liver first approach in CRLM. The 15 papers included encompass the three main strategies in the treatment of CRLM; colorectal first, liver first, and simultaneous resection. However, we specifically focused our primary and secondary endpoints on the colorectal first versus liver first strategies. We included 11 638 patients from retrospective studies with no RCTs available. We were able to demonstrate a statistically significant benefit in the overall survival of patients undergoing the CRf strategy over the Lf strategy in the 1st year after resection; however, this benefit was eliminated in the 3‐ and 5‐ year follow‐up periods.

Following the second primary endpoint, there was a clearer benefit in the disease‐free survival of patients undergoing the CRf strategy over the Lf, which was demonstrated in both 3‐ and 5‐ year follow‐ups on the 3 studies that measured this. A previous meta‐analysis published in 2020 by Magouliotis [13] et al included 10 studies with a total of 3656 patients comparing the same primary endpoints along with 30‐, 90‐day mortality, length of stay, and major morbidity: demonstrating no statistically significant difference in any endpoint. Similar limitations to this study were present, with a lack of RCTs and heterogeneous, retrospective evidence. Another previous systematic review and meta‐analysis conducted by Winter et al [35] included 3,605 patients and compared similar endpoints of overall survival and 30‐day mortality, and again did not show any statistical difference in any endpoint. Similarly, another systematic review and meta‐analysis conducted Roberts et al. [36], which also compared a simultaneous resection strategy along with CRf and Lf failed to show any statistically significant benefit in any method in overall survival and morbidity. Interestingly, as this was a network analysis, rankograms of cumulative ranking probabilities were created, favoring the Lf approach over the CRf and simultaneous approaches. A recent retrospective cohort study by Mantke et al. [37] published in 2025 included 209 patients and showed a statistically significant overall survival benefit (OS of 68% vs. 53%, respectively) in a staged approach where the colorectal primary is resected first, followed by liver resection, consistent with our findings.

Our inclusion of more recent papers and a significantly higher *n* number compared to previous meta‐analyses, along with a statistically significant benefit demonstrated in DFS and OS, may indicate that although both strategies are feasible and safe, there may be a benefit in CRf over the Lf strategy. A reason as to why this effect was demonstrated compared to previous meta‐analyses was likely that higher statistical power was required to demonstrate it. Within the study context, this is unsurprising due to the heterogeneity of available evidence, retrospective and mostly per‐protocol analysis, along with the high number of confounding variables found when analyzing such data.

The statistically significant overall survival benefit in the 1st year after resection can potentially be explained by the emergent complications that are seen in colorectal cancer, such as obstruction or perforation, which have generally higher associated morbidity and mortality than complications of liver metastases, along with a lower response to chemotherapy [38, 39, 40]. Early primary resection will thus lower the risk of these complications arising, whereas the liver‐first approach will not, increasing the risk of bias. Previous data suggest that about one out of three cases planned for Lf approach do not proceed to resection of the primary tumor [41]. At this point, it must also be stated that most Lf patients have a higher burden of liver disease. Furthermore, this subgroup of patients has often co‐occurrence of RAS (often *KRAS* or *NRAS*) and TP53 mutations, a high‐risk molecular profile that exhibits a higher likelihood of relapse following surgical intervention, likely by enhancing chemoresistance [42].

In terms of disease‐free survival, which was sustained over the 5 years of follow‐up, oncologic changes in the metastatic tumor milieu may offer an explanation. It is known that the immune tumor microenvironment between the colorectal primary and the liver metastasis vary, with a higher proportion of immunosuppressive macrophages found in the metastatic portion. This higher expression of CD68^−^CD163^+^ macrophages may be related to the reduced efficacy of immunotherapy seen in liver metastases, which could contribute to this significant difference [43]. However, another potential explanation is that from the included studies, patients who underwent the Lf strategy had a higher degree of hepatic tumor burden and were offered the operation for downstaging. We can thus potentially infer that higher hepatic tumor burden likely represents a less prognostically favorable primary phenotype, which could potentially negatively influence disease‐free survival as demonstrated in our data. In addition, a large issue regarding staged resections is the use of adjuvant and/or neoadjuvant chemotherapy for liver metastases. The majority of our included studies reported neoadjuvant chemotherapy use in the Lf groups, which has been used historically in CRLM for the downstaging of “borderline resectable” lesions with a high hepatic tumor burden [44]. However, the authors also report that there is no clear definition or consensus of “borderline” tumors; therefore, neoadjuvant chemotherapy use is inconsistent amongst centers, further confounding any conclusions. Moreover, for resectable CRLM there is conflicting evidence regarding the use of neoadjuvant chemotherapy, with upfront surgery demonstrating superiority in OS in some studies and neoadjuvant chemotherapy demonstrating superiority in DFS [45, 46, 47, 48]. Despite this, surgery remains the only curative option for these patients [49].

Another controversial topic in staged resections is the timing, with no clear consensus or guidelines. Historically, in CRf approaches, liver resection would be performed 2–3 months later with or without interval chemotherapy [50]; however, this was based on expert opinion.

An important further source of confounding is that not all included papers controlled for rectal versus colonic primaries. The management of colon cancer versus low, middle, and high rectal cancer differs vastly, with different chemotherapy regimens and use of radiotherapy. Due to this, performing subgroup analysis for rectal versus colon cancer was deemed not feasible. A significant challenge in patient selection is that the site of the primary tumor significantly influences resection strategy, rather than a purely oncological evidence‐based decision. A liver‐first approach appears more feasible in rectal tumors compared to colonic tumors due to the established benefit of neoadjuvant chemoradiotherapy, which does not always necessitate resection [51] of the primary; however, resection of the liver metastases will provide a survival benefit [49]. Additionally, extended chemotherapy courses for rectal primaries may increase peri‐operative risk for the second resection, along with chemotherapy‐induced hepatotoxicity and alterations in function influencing the required future liver remnant (FLR). Conversely, a colorectal approach will be significantly favored in patients presenting acutely with complications such as obstruction, with the primary operation done in the acute phase or after temporizing measures such as stenting.

As discussed, the major limitations of this study are related to the limitations of the included literature. A pool of relatively limited in number, retrospective cohort studies and no RCTs poses a higher risk of bias and heterogeneity within the analysis. Furthermore, a lack of standardization and definitions means that terms such as “resectable” and “borderline” are varied and inconsistent, so drawing sound conclusions becomes more challenging. Furthermore, the differing use of neoadjuvant chemotherapy, size and location of metastases, and overall hepatic tumor burden score lead to grouping together of patients with significantly differing prognostic indicators from the start. Additionally, the same grouping will occur with patients receiving different chemotherapeutic regimens and at different timings. Moreover, the lack of control for colon versus rectal primaries may also influence our endpoints due to the significant prognostic differences associated with their respective staging, along with the neoadjuvant use of radiotherapy and chemotherapy protocols. As this meta‐analysis focused on staged resections, another limitation was the lack of reported failure‐to‐proceed to the second stage rate. All but three included studies were conducted as per protocol analysis, which does not take into consideration patients who were unable to tolerate the second resection for a multitude of reasons. This is something that the MDT will have to take into consideration when discussing resection strategy.

Still, our meta‐analysis provides the most up‐to‐date evidence regarding staged resections for CRLM and was, to our knowledge, the first to show a statistically significant benefit of the CRf approach compared to the Lf. Compared to previous meta‐analyses, our higher number of patients, clear primary endpoints of OS and DFS, use of three different databases by two independent authors, and control for heterogeneity have likely helped to reach these conclusions.

## 7 Conclusion

There is a statistically significant benefit in both OS and DFS in patients undergoing the CRf strategy compared to the Lf. No difference was found in 90‐day mortality, major morbidity, and failure to proceed to the second stage. However, it is clear that MDTs should tailor each strategy to each patient, as hepatic tumor burden, complications, and extent and location of primary disease, along with tolerance of chemotherapy, heavily influence this choice. The findings of this meta‐analysis, which is based on retrospective data, should be interpreted with caution.

## Funding

The authors have nothing to report.

## Conflicts of Interest

The authors declare no conflicts of interest.

## Statement of Originality

This paper attempts to elucidate the optimal resection sequence in patients with simultaneous colorectal liver metastasis, colorectal first versus liver first approach. This systematic review and meta‐analysis reflects the latest literature and, to our knowledge, the first paper to suggest a statistically significant benefit of colorectal first versus liver first.

## Synopsis

Optimal sequence of resection in simultaneous colorectal liver metastases remains controversial. This systematic review and meta‐analysis included 15 studies comparing overall, disease‐free survival, major morbidity, and mortality outcomes. One‐year overall and disease‐free survival rates were found to be significantly higher in the colorectal first group.

## Supporting information

Supporting File 1

Supporting File 2

## Data Availability

The data that support the findings of this study are available from the corresponding author upon reasonable request.
