# Supplementary material for: Systematic Review and Meta‐Analysis of Resection Sequence in Synchronous Colorectal Liver Metastasis: Primary vs. Liver First
Source: J Surg Oncol. 2026 Jun 15;134(3):444–54. doi: 10.1002/jso.70309 (PMC13532932; doi:10.1002/jso.70309)
Supplement: Supplementary file 1 [file JSO-134-444-s001.docx]

**Identification of studies via databases and registers**

Records removed *before screening*:

Duplicate records removed (n = 3653)

Records identified:

PubMed (n = 3507)
Google Scholar (n = 56)

Cochrane trials (n = 24192)

**Identification**

Records screened

(n = 20539)

Records excluded

(n = 20510)

Reports sought for retrieval

(n = 29)

Reports not retrieved

(n = 0)

**Screening**

Reports assessed for eligibility

(n = 29)

Reports excluded:

Review (n = 10)

Overlapping data (n = 2)

Letter to the Editor (n = 1)

Missing Follow up data (n=1)

Studies included in review

(n = 15)

**Included**

Source: Page MJ, et al. BMJ 2021;372:n71. doi: 10.1136/bmj.n71.

This work is licensed under CC BY 4.0. To view a copy of this license, visit <https://creativecommons.org/licenses/by/4.0/>
