# Supplementary material for: Systematic Review and Meta‐Analysis of Resection Sequence in Synchronous Colorectal Liver Metastasis: Primary vs. Liver First
Source: J Surg Oncol. 2026 Jun 15;134(3):444–54. doi: 10.1002/jso.70309 (PMC13532932; doi:10.1002/jso.70309)
Supplement: Supplementary file 2 [file JSO-134-444-s002.docx]

**Supplementary material**
